# Asymmetrically dividing *Drosophila* neuroblasts utilize two spatially and temporally independent cytokinesis pathways

**DOI:** 10.1038/ncomms7551

**Published:** 2015-03-20

**Authors:** Michaela Roth, Chantal Roubinet, Niklas Iffländer, Alexia Ferrand, Clemens Cabernard

**Affiliations:** 1 Biozentrum, University of Basel, Klingelbergstrasse 50-70, CH-4056 Basel, Switzerland; 2 Imaging Core Facility (IMCF), Biozentrum, University of Basel, Klingelbergstrasse 50-70, CH-4056 Basel, Switzerland

## Abstract

Precise cleavage furrow positioning is required for faithful chromosome segregation and cell fate determinant distribution. In most metazoan cells, contractile ring placement is regulated by the mitotic spindle through the centralspindlin complex, and potentially also the chromosomal passenger complex (CPC). *Drosophila* neuroblasts, asymmetrically dividing neural stem cells, but also other cells utilize both spindle-dependent and spindle-independent cleavage furrow positioning pathways. However, the relative contribution of each pathway towards cytokinesis is currently unclear. Here we report that in *Drosophila* neuroblasts, the mitotic spindle, but not polarity cues, controls the localization of the CPC component Survivin. We also show that Survivin and the mitotic spindle are required to stabilize the position of the cleavage furrow in late anaphase and to complete furrow constriction. These results support the model that two spatially and temporally separate pathways control different key aspects during asymmetric cell division, ensuring correct cell fate determinant segregation and neuroblast self-renewal.

## Abstract (web summary)

In asymmetrically dividing cells, both spindle-dependent and spindle-independent cleavage furrow positioning pathways are involved in cytokinesis. Here the authors find that Survivin and the mitotic spindle are required to stabilize the position of the cleavage furrow and to complete cytokinesis in *Drosophila* neuroblasts.

## Graphical abstract

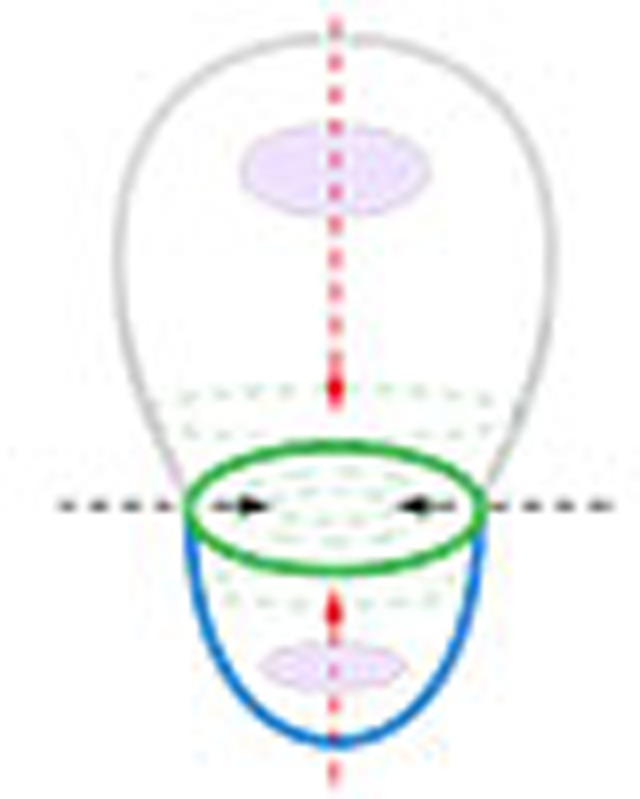

The mitotic cycle of symmetrically and asymmetrically dividing cells completes with cytokinesis, the process that physically separates two sibling cells, ensuring the proper partitioning of the nuclear and cytoplasmic contents at the end of cell division. The correct placement of the contractile ring is of fundamental importance, since inaccurate positioning can lead to the partitioning of both nuclei into one daughter cell, causing tetraploidy, tumour formation and cancer^1^. During asymmetric cell division, mispositioning of the cleavage furrow can also result in defective segregation of cell fate determinants and in changes in cell fate and cell behaviour^2^,^3^.

In most metazoan cells, the positional cues for contractile ring placement and assembly originate from the mitotic spindle in the form of the centralspindlin complex^4^. This complex is evolutionary conserved and is composed of the mitotic kinesin-like protein 1 (MKLP1) orthologue Pavarotti (Pav in *Drosophila*; Zen-4 in *C. elegans*) and Tumbleweed (Tum in *Drosophila*; MgcRacGAP in vertebrates and CYK-4 in *C. elegans*; reviewed in refs 4, 5). Current models propose that Pav/MKLP1 travels along stable cortical microtubules (MTs), delivering Tum to the cell equator where it activates the RhoGEF Pebble (Pbl in *Drosophila*; ECT2 in vertebrates and LET-21 in *C. elegans*)^6^,^7^,^8^. Pbl in turn activates the small GTPase Rho1, recruiting contractile ring components to the cleavage furrow (reviewed in refs 4, 5). The non-cortical fraction of the centralspindlin complex is localized on the central spindle, an array of overlapping antiparallel microtubules that is formed during early anaphase. Centralspindlin’s localization to the central spindle is controlled by the chromosomal passenger complex (CPC), consisting of Aurora B kinase, the inner centromere protein (INCENP), Survivin and Borealin. Both centralspindlin and the CPC are required for central spindle assembly. It has been suggested that the CPC is involved in cleavage furrow positioning since its component INCENP has been found to be localized to the presumptive cleavage furrow prior to the contractile ring component non-muscle Myosin II (Myosin hereafter)^9^. However, CPC-mediated furrow positioning could indirectly occur through Rho1 activation^10^. The role of the CPC as a major mitosis regulator is undisputed, but its function during cytokinesis in general and asymmetric cell division in particular is less clear^10^.

There is also accumulating evidence for the existence of spindle-independent cleavage furrow positioning mechanisms^11^,^12^,^13^,^14^. For instance, *Drosophila* neuroblasts utilize a cortical polarity-dependent pathway for contractile ring placement^11^,^15^. Neuroblasts are the precursors of the fly’s central nervous system and undergo repeated rounds of asymmetric cell divisions, generating differentiating ganglion mother cells while self-renewing the neuroblast at the same time^16^. During asymmetric cell division, the accurate positioning of the cleavage furrow is very important, since misplaced furrows compromise precise cell fate determinant segregation, affecting neuroblast homoeostasis and differentiation^2^. The cortical polarity-dependent pathway induces asymmetric Myosin localization; in contrast to symmetrically dividing cells, Myosin clears first from the apical neuroblast cortex shortly after anaphase onset. On the basal cortex, however, Myosin is removed only later in anaphase. Genetic and chemical spindle ablation and rotation experiments demonstrated that this symmetry-breaking event is independent of MTs (spindle independent), but requires the polarity proteins Discs large 1 (Dlg1) and partner of inscuteable (Pins; AGS3/LGN in vertebrates; polarity dependent)^11^,^15^. This asymmetric Myosin distribution allows for unequal cortical expansion, pushing the cleavage furrow towards the basal cortex^15^, a mechanism that is conserved in the *C. elegans* Q-neuroblast lineage^12^. The molecular mechanisms of this polarity-dependent furrow-positioning pathway are currently not understood.

In neuroblasts, also in other cells containing two cytokinesis pathways, the function and relative contribution of each pathway in cytokinesis is unclear. Thus, in order to elucidate the exact role of the spindle-dependent pathway in asymmetrically dividing neuroblasts, a component needs to be identified, acting only in the spindle-dependent but not in the polarity-dependent pathway. Recently, it was shown that the centralspindlin component Pav also localizes in a spindle-independent manner^11^, suggesting that the centralspindlin complex could be shared between the polarity-dependent and spindle-dependent pathways. However, whether this also applies to the CPC remains to be tested.

Here we use live imaging, super-resolution microscopy, photoconversion and fluorescence recovery after photobleaching (FRAP) experiments to carefully monitor the dynamic localization of the CPC during asymmetric cell division. We find that the CPC component Survivin only acts in the spindle-dependent pathway and plays an important role in stabilizing basal cleavage furrow positioning, MT bundling and cleavage furrow constriction but is dispensable for asymmetric Myosin localization. With Survivin we thus identified a specific spindle-dependent cytokinesis component, not intersecting with the polarity-dependent pathway. The data presented here extend our model, proposing that asymmetrically dividing *Drosophila* neuroblasts employ two spatially and temporally separable cytokinesis pathways. The polarity-dependent pathway specifically induces asymmetric Myosin localization, shifting the cleavage furrow to the basal position, but is dispensable for cleavage furrow constriction. The spindle-dependent pathway is using the CPC to stabilize the positioning of the basal cleavage furrow and subsequently completes cleavage furrow constriction.

## Results

### Myosin precedes Survivin's cleavage furrow recruitment

To understand the dynamic localization of the CPC during asymmetric cell division, we started our analysis with time lapse imaging experiments of the CPC component Survivin in *Drosophila* neuroblasts. In symmetrically dividing mammalian cells, the CPC very dynamically relocalizes from chromosomes to spindle midzone microtubules and to the cleavage furrow (reviewed in refs 10, 17). However, CPC localization dynamics has not been analysed in asymmetrically dividing neuroblasts and it is currently unknown whether the polarity-dependent cytokinesis pathway or the mitotic spindle controls the CPC.

**Figure 1:**
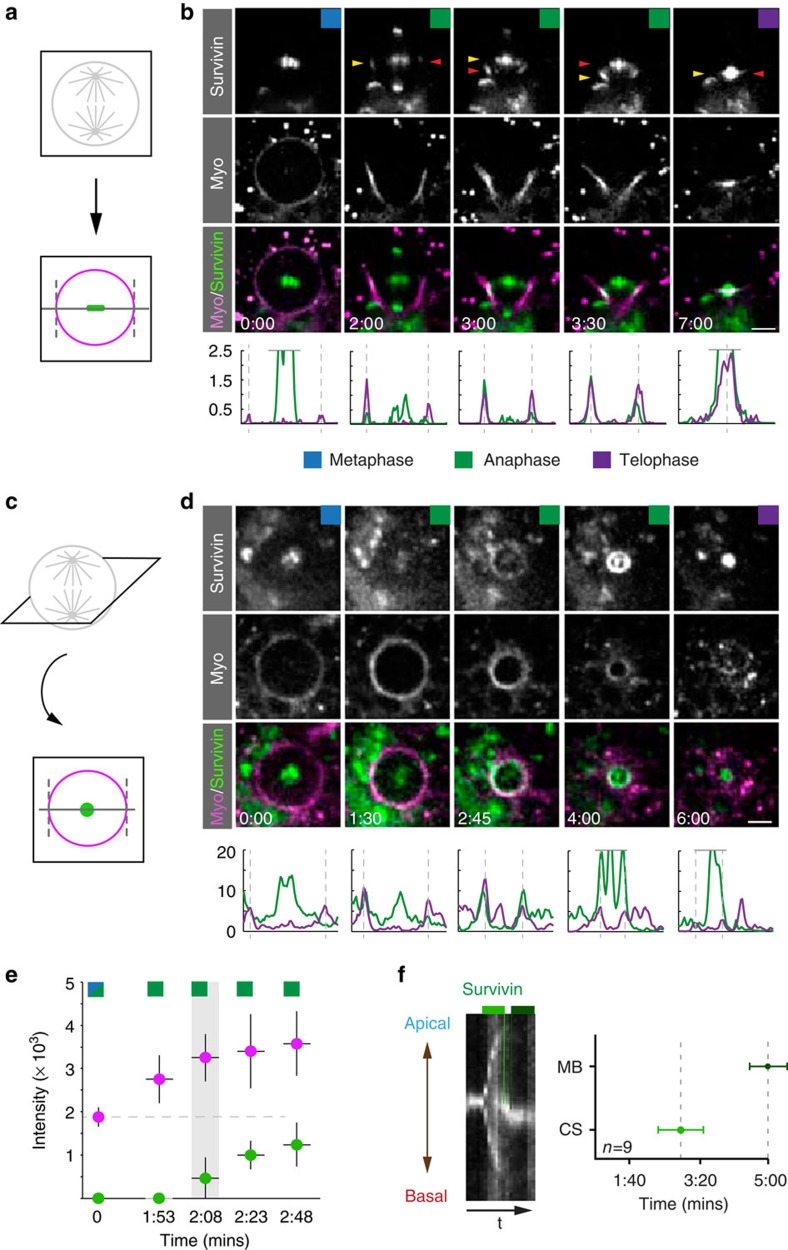
Myosin is localized to the cleavage furrow prior to Survivin. (**a**) Third instar larval neuroblasts were imaged along the *xy* axis (horizontal) or (**c**) en-face (vertical); intensity measurements were performed along a line through the midplane or at the level of the ingressing cleavage furrow, respectively. (**b**) and (**d**) Image sequence of a wild-type neuroblast expressing Survivin::GFP (green in overlay) and Sqh::mCherry (Myo; magenta in overlay). Intensity plots (Survivin, green; Myosin, magenta) are shown underneath the corresponding images. Intensity curves exceeding the scale were cut off (horizontal grey line). The vertical dashed lines refer to the position of the cortex. For all subsequent figures, coloured squares refer to the mitotic stage indicated below. Yellow and red arrowheads highlight the level of Survivin on the contractile ring and the central spindle, respectively. (**e**) Fluorescence intensity was measured for Survivin::GFP and Sqh::mCherry to determine the time when Survivin (green) reaches the cortical position of the future cleavage furrow in relation to Myosin (magenta; *n*=7). (**f**) Kymograph along the division axis. Note that towards the end of mitosis, Survivin shifts basally. The light green line and measurement point refer to Survivin appearing at the central spindle (CS). The dark green line and measurement point indicate Survivin shifting basally to the midbody (MB). Time in min:s; scale bar, 5 μm.

To this end, we used a functional genomic Survivin::GFP construct^18^ and performed live imaging experiments in third instar larval neuroblasts (see Methods). We found that Survivin localizes to centromeres/kinetochores in prometaphase and metaphase, respectively, before translocating to the cell poles on separating chromosomes. During anaphase, Survivin also appeared on interdigitating microtubules that form the central spindle, becoming more enriched as anaphase progressed and moved into telophase. At the onset of furrowing, Survivin can be detected in close proximity to the neuroblast cortex, either at the cell equator, but mostly in a slightly basally shifted position (Fig. 1a–d; Supplementary Fig. 1a,b; Supplementary Movies 1 and 2).

Since it has been reported that the CPC component INCENP precedes Myosin accumulation at the equatorial cortex, suggesting a putative role for the CPC in cleavage furrow positioning and contractile ring assembly^9^, we wondered whether this is also the case for Survivin in asymmetrically dividing neuroblasts. To this end, we imaged Sqh::mCherry^19^ (Myosin’s regulatory subunit fused to mCherry; Myosin/Myo hereafter) together with Survivin::GFP^18^ and measured Survivin’s appearance at the cell cortex in relation to Myosin. Cortical Survivin becomes detectable ~2 min after anaphase onset (mean=128 s; s.d.=22 s; *n*=7; Fig. 1e). Myosin accumulates at the cortex uniformly already during prometaphase and metaphase, preceding Survivin’s cortical localization (Fig. 1b,d,e). However, cortical Survivin accumulation coincides with the onset of furrowing, and we detected a concomitant increase of both Myosin and Survivin (Fig. 1d,e).

We used kymograph analysis to investigate Survivin’s association with the central spindle in more detail. We measured that ~2–3 min after anaphase onset (mean=172 s; s.d.=43 s; *n*=9), Survivin accumulated right at the center of the mitotic spindle, coinciding with the bundling of microtubules (Fig. 1f). At this time point, the central spindle is still symmetric but apical cortical expansion already started, shifting the cleavage furrow to a basal position^11^,^15^. Survivin associated with the central spindle is thus positioned slightly apically in relation to the ingressing cleavage furrow (see Fig. 1b, time point 3:00–3:30; Fig. 1f). By telophase, ~5 min after anaphase onset (mean=297 s; s.d.=36 s; *n*=9), the central spindle and with it central spindle-associated Survivin shifted basally so that the ingressing cleavage furrow is perfectly centred on the Survivin spot that marks the central spindle (Fig. 1b; time point 7:00). Taken together, we found that in *Drosophila* neuroblasts, kinetochore-localized Survivin splits up into three distinct populations: a kinetochore-associated fraction during chromosome separation; a central spindle pool; and a cortex-associated Survivin, colocalizing with Myosin. These measurements reveal that compared with mammalian cells, Survivin localizes in a similar spatial localization pattern. However, Myosin is already at the cell cortex when Survivin appears. These results thus raise three important questions: what is the origin of these three distinct Survivin pools? By what mechanism is Survivin relocalized? Does the polarity-dependent pathway, known to act in asymmetrically dividing neuroblasts, influence Survivin’s localization?

### Cortical Survivin originates exclusively from kinetochores

**Figure 2:**
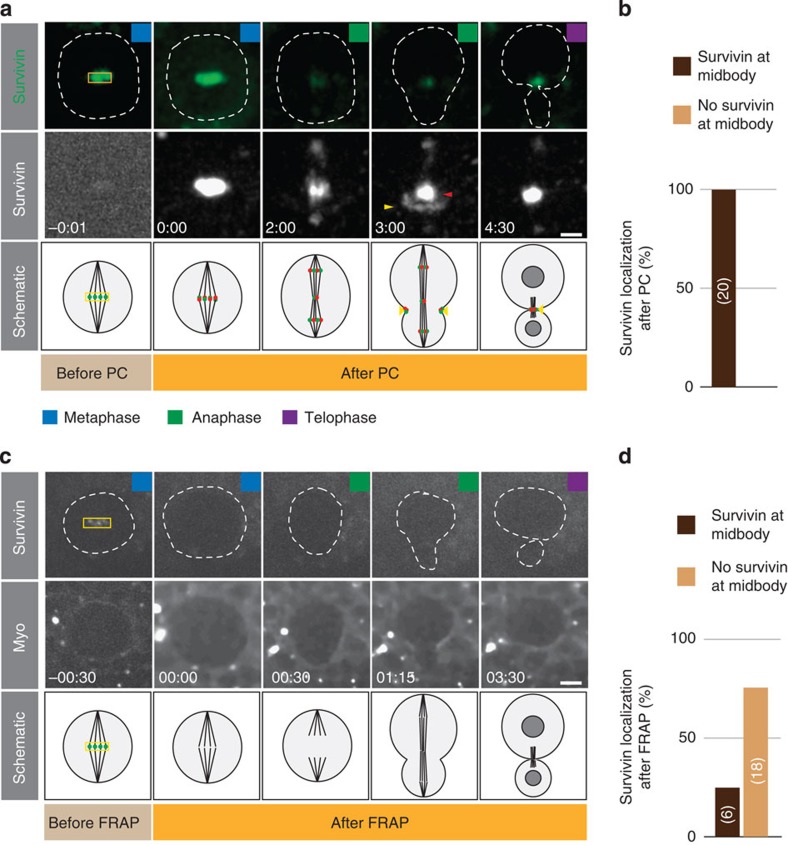
Cortical Survivin originates from the kinetochore. (**a**) Image sequence of a representative wild-type neuroblast expressing Survivin::mDendra2. Top row shows unconverted (green), middle row photoconverted (white) Survivin::mDendra, respectively. Photoconversion (PC) was performed on the metaphase plate (yellow square). Red and yellow arrowheads refer to photoconverted Survivin at the central spindle and contractile ring, respectively. Schematic representation shown below. (**b**) Quantification of the photoconversion experiment. Number of scored neuroblasts is highlighted in bars. (**c**) FRAP experiments performed at the metaphase plate (yellow square) on Survivin::GFP (top row). Sqh::mCherry (Myo) was imaged to visualize the cortex and cleavage furrow. Dashed lines outline the neuroblast. (**d**) Quantification of the FRAP experiment. Number of scored neuroblasts is highlighted in bars. Coloured squares refer to the cell cycle stage as defined in Fig. 1. Time in min:s; scale bar, 5 μm.

In order to address these questions, we first explored the origin of the three Survivin pools and the mechanism by which they reach these subcellular locations. For instance, cleavage furrow-associated Survivin could originate from the metaphase centromere/kinetochore-bound fraction. Alternatively, Survivin could be recruited from the cytoplasm to specific subcellular sites during anaphase. To distinguish between these two scenarios, we tagged Survivin with the photoconvertable fluorescent protein mDendra2 and performed *in vivo* pulse-chase labelling experiments (see Methods). Photoconverting Survivin at the metaphase midplane with a short pulse of ultraviolet light resulted in an immediate and robust change in emission that could be traced from metaphase onwards until telophase. We found that photoconverted Survivin was localized on separating kinetochores, the central spindle population and in the furrow region (Fig. 2a,b), indicating that the Survivin molecules accumulating at the midbody by telophase, originated from the kinetochores in metaphase (100%, *n*=20). However, the possibility remained that newly synthesized or cytosolic Survivin relocalized to the cleavage furrow. We tested this using FRAP and bleached Survivin::GFP at the metaphase plate. In most cases, no signal was detectable at the midbody after complete quenching of Survivin::GFP (75%, *n*=24; Fig. 2c,d). Similarly, photoconversion experiments targeting the cytoplasm did not result in any detectable signal at the metaphase plate nor at the midbody in telophase (100%; *n*=6; Supplementary Fig. 2a,b). Thus, we can conclude the following: the three Survivin populations all originate from metaphase kinetochore-bound Survivin and that from anaphase onset onwards no new Survivin molecules are recruited to kinetochores, the contractile ring and the central spindle. Furthermore, these data suggest that Survivin turn-over rates are very low from metaphase onwards until telophase.

### Survivin’s relocalization depends on the mitotic spindle

**Figure 3:**
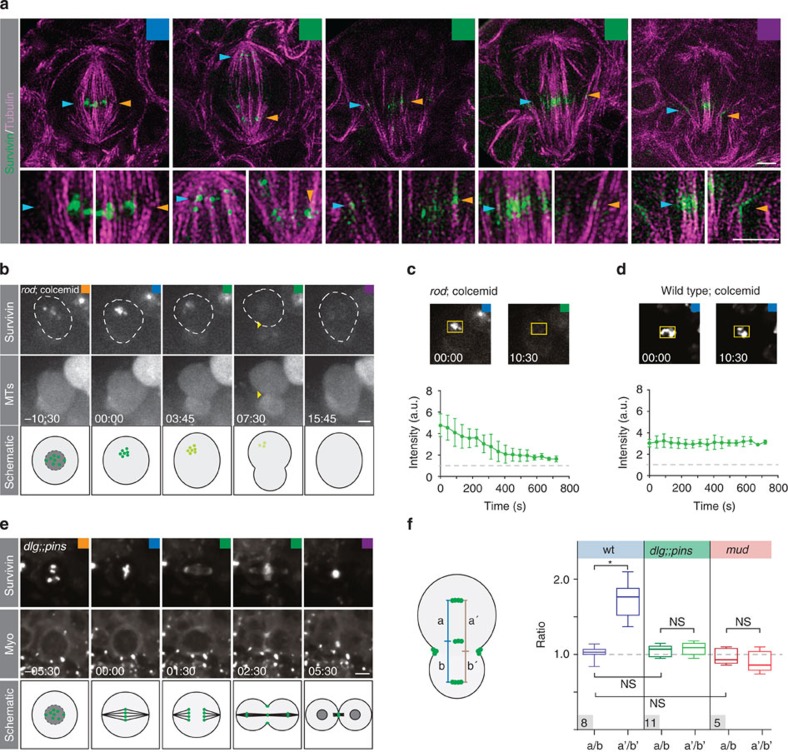
Survivin’s relocalization depends on the spindle and anaphase entry. (**a**) 3D-SIM images of third instar larval brain wild-type neuroblasts, expressing Survivin::GFP (green) and stained with α-Tubulin (magenta). High-magnification pictures of areas of interest (blue and orange arrows) are shown below the overview pictures. Coloured arrowheads highlight Survivin clusters in association with microtubules. (**b**) Image sequence of a representative *rod*^*H4.8*^ mutant neuroblast treated with colcemid, imaged with Survivin::GFP (top row) and the spindle marker mCherry::Jupiter (middle row). Schematic below; green dots represent Survivin molecules fading away. (**c**) Intensity measurements of Survivin::GFP in *rod*^*H4.8*^ and (**d**) wild-type neuroblasts treated with colcemid. The graph shows average intensity. Error bars correspond to s.d. (wt; *n*=10, *rod* and colcemid; *n*=8). (**e**) Image sequence of a representative *dlg*^*m52*^*;;pins*^*P89*^ mutant neuroblast expressing Survivin::GFP (top row) and Sqh::mCherry (Myo; second row), dividing symmetrically. (**f**) Measured distance ratios between the indicated Survivin pools in wild-type, *dlg*^*m52*^*;;pins*^*P89*^ and *mud*^*4*^ mutant neuroblasts. Number of scored cells are indicated in the grey box. Asterisk (*) denotes statistical significance. *P*=0.000023 (two-sample unequal variance *t*-test). NS, not significant; *P*>0.01 (based on two-sample equal or unequal variance *t*-test). Time in min:s; scale bar, 2 μm in panel (**a**) and 5 μm in all subsequent panels. wt, wild type.

Next, we investigated how kinetochore-bound Survivin reaches the contractile ring. Previous reports suggest that Survivin’s dynamic redistribution depends on microtubules (reviewed in ref. 10). To test this, we first used super-resolution microscopy to image larval brains expressing Survivin::GFP and stained with anti-αTubulin (see Methods). Survivin::GFP accurately reflects Survivin’s localization but provides better signal-to-noise ratio compared with the anti-Survivin antibody^18^ (Supplementary Fig. 3a,b). We found that Survivin clusters generally colocalized with microtubule fibres. For instance, Survivin was associated with MTs forming the central spindle. Furthermore, we detected Survivin clusters, colocalizing with MTs extending towards the furrow region in anaphase and telophase cells (Fig. 3a).

To test whether microtubules are required for Survivin’s accumulation at the contractile ring, we chemically ablated the mitotic spindle using colcemid (see Methods) in neuroblasts devoid of the spindle-assembly checkpoint (using *rough deal (rod)* mutants; see Methods and refs 11, 20), driving neuroblasts into anaphase despite the lack of a mitotic spindle. We confirmed that *rod* mutant neuroblasts treated with colcemid and expressing the spindle marker mCherry::Jupiter^2^, lacked discernable spindles and initiated constriction, albeit cytokinesis did not complete^11^. Under these conditions, Survivin was still associated with chromosomes during metaphase but as the cell entered anaphase, chromatin-bound Survivin dissipated into the cytoplasm. However, Survivin failed to relocalize to the cell poles, the central spindle and the cell cortex. Also, we did not find any Survivin localized to the forming cleavage furrow (100%; *n*=25; Fig. 3b,c). However, if microtubules were depolymerized in neuroblasts containing an intact spindle-assembly checkpoint, Survivin remained localized to chromatin (100%; *n*=25; Fig. 3d).

These results suggest that the mitotic spindle is required for Survivin’s redistribution. We thus tested whether changes in spindle geometry will also affect Survivin’s central spindle and cortical localization. Approximately 15% of *mud* mutant and essentially all *dlg;;pins* double-mutant neuroblasts contain symmetric spindles^21^,^22^. Indeed, we found that in *mud* and *dlg;;pins* mutant neuroblasts, the position of Survivin, colocalizing with Myosin on the ingressing cleavage furrow, perfectly matched up with central spindle-associated Survivin (Fig. 3e,f), which is not the case in wild-type neuroblasts (Figs 1b,f and 3f). On the basis of these results, we conclude that entry into the anaphase is required to unload Survivin from centromeres/kinetochores. We further conclude that microtubules are required for Survivin’s dynamic relocalization and that spindle geometry determines the position of Survivin’s central spindle-associated and cleavage furrow pool.

### Survivin localizes independently of the polarity pathway

**Figure 4:**
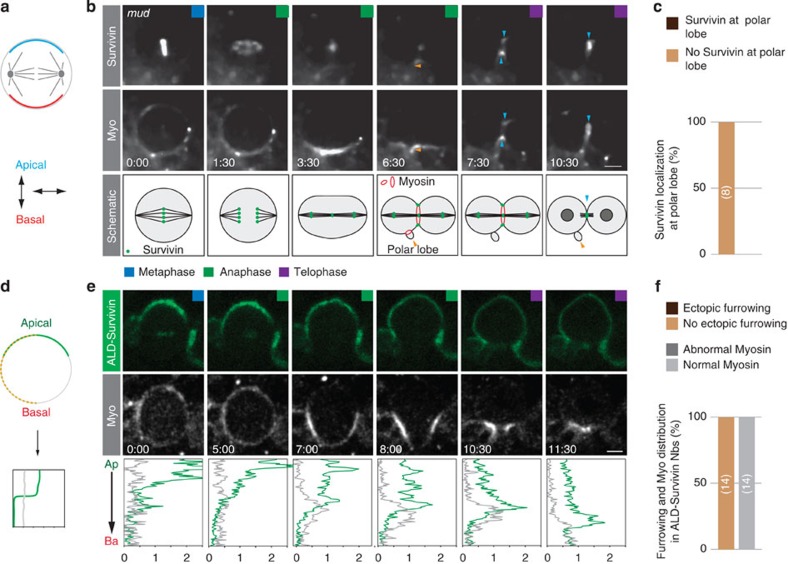
Survivin’s localization is independent of the polarity pathway. (**a**) Schematic representation of the genetic spindle rotation experiment to uncouple the orientation of the mitotic spindle in relation to the neuroblast intrinsic polarity axis (apical, blue; basal, red). (**b**) Image sequence of a representative *mud*^*4*^ mutant neuroblast expressing Survivin::GFP (top row) and Sqh::mCherry (Myo; second row), dividing symmetrically and forming a polar lobe (06:30; orange arrowhead). Blue arrowheads highlight Survivin localization at the spindle-induced furrow. (**c**) Quantification of Survivin appearance at polarity-dependent cleavage furrow (polar lobe). Number of cells scored is highlighted in bars. (**d**) Survivin was ectopically localized at the apical neuroblast cortex (green); intensity measurements were performed along the dashed yellow line from apical to basal. (**e**) Third instar larval neuroblast expressing ALD-Survivin::EGFP (top row; green) and Sqh::mCherry (bottom row; white). Intensity measurements (green, ALD-Survivin::EGFP; grey, sqh::mCherry) are shown below. (**f**) Quantification of ectopic furrowing and Myosin distribution. Number of cells scored is highlighted in bars. Ap, apical; Ba, basal. Time in min:s; scale bar, 5 μm.

We next wanted to test whether Survivin can also get recruited to spindle-independent furrows. For instance, we used *mud* mutants to uncouple the mitotic spindle from the neuroblast intrinsic polarity axis^23^,^24^,^25^ (Fig. 4a), inducing a spatially and temporally separate polarity-induced cleavage furrow on the basal cortex, manifested in the formation of a polar lobe^11^. All neuroblasts developing a polarity-dependent cleavage furrow failed to localize Survivin to this ectopic location (100%; *n*=8; Fig. 4b,c). However, as soon as the spindle-dependent cleavage furrow forms, Survivin can be found to colocalize with Myosin at the spindle-dependent furrow (Fig. 4b). Thus, this experiment suggests that Survivin is not recruited to the polarity-dependent cleavage furrow and is dispensable for the initiation of furrowing.

Finally, we asked whether Survivin is sufficient to induce cleavage furrow positioning and furrow constriction. To this end, we ectopically targeted Survivin tagged with EGFP to the apical neuroblast cortex using the apical localization domain (ALD) from inscuteable (Insc^26^; Fig. 4d); wild-type neuroblasts never show Survivin accumulation at the apical cortex, providing us with a reliable readout for Myosin recruitment and ectopic furrowing. We co-expressed ALD-Survivin::EGFP with Myosin (sqh::mCherry^19^) and found robust localization of ALD-Survivin::EGFP to the apical neuroblast cortex from prophase onwards. We could also detect weak centromere/kinetochore localization during metaphase and the central spindle in telophase, suggesting that a small fraction of ALD-Survivin::EGFP is not recruited to the apical neuroblast cortex. However, despite the presence of Survivin on the apical cortex, (Supplementary Fig. 4a) we failed to observe ectopic furrow formation. Although Myosin partially overlaps with ALD-Survivin—especially at metaphase—no ectopic Myosin recruitment was observed (Fig. 4e,f; Supplementary Movie 3). Similarly, AuroraB kinase did not colocalize with ALD-Survivin, but was localized indistinguishable from wild-type neuroblasts (Supplementary Figs 3c and 4b). These experiments suggest that cortical Survivin is neither sufficient to recruit AuroraB and Myosin to the cortex nor to induce cleavage furrow positioning and formation. Taken together, we conclude that Survivin’s localization strictly depends on the mitotic spindle, following spindle geometry and architecture. We further conclude that polarity cues in general, and Dlg and Pins in particular, are dispensable for Survivin’s central spindle and cortical localization.

### Survivin is required for contractile ring constriction

The data presented so far strongly suggest that Survivin is a specific component of the spindle-dependent cytokinesis pathway. Thus, *survivin* mutants provide a useful tool to explore the function of the spindle-dependent cytokinesis pathway during asymmetric neuroblast division. However, since the CPC is associated with many functions during mitosis, complete loss of function alleles cannot be used to specifically address Survivin’s function in cytokinesis. To circumvent this caveat, we utilized a temperature-sensitive separation-of-function allele of *survivin*, specifically disrupting CPC localization and function from anaphase onwards but not before^18^. *scpo* mutant neuroblasts show cytokinesis defects, but it is unclear which aspect of cytokinesis is compromised^18^.

**Figure 5:**
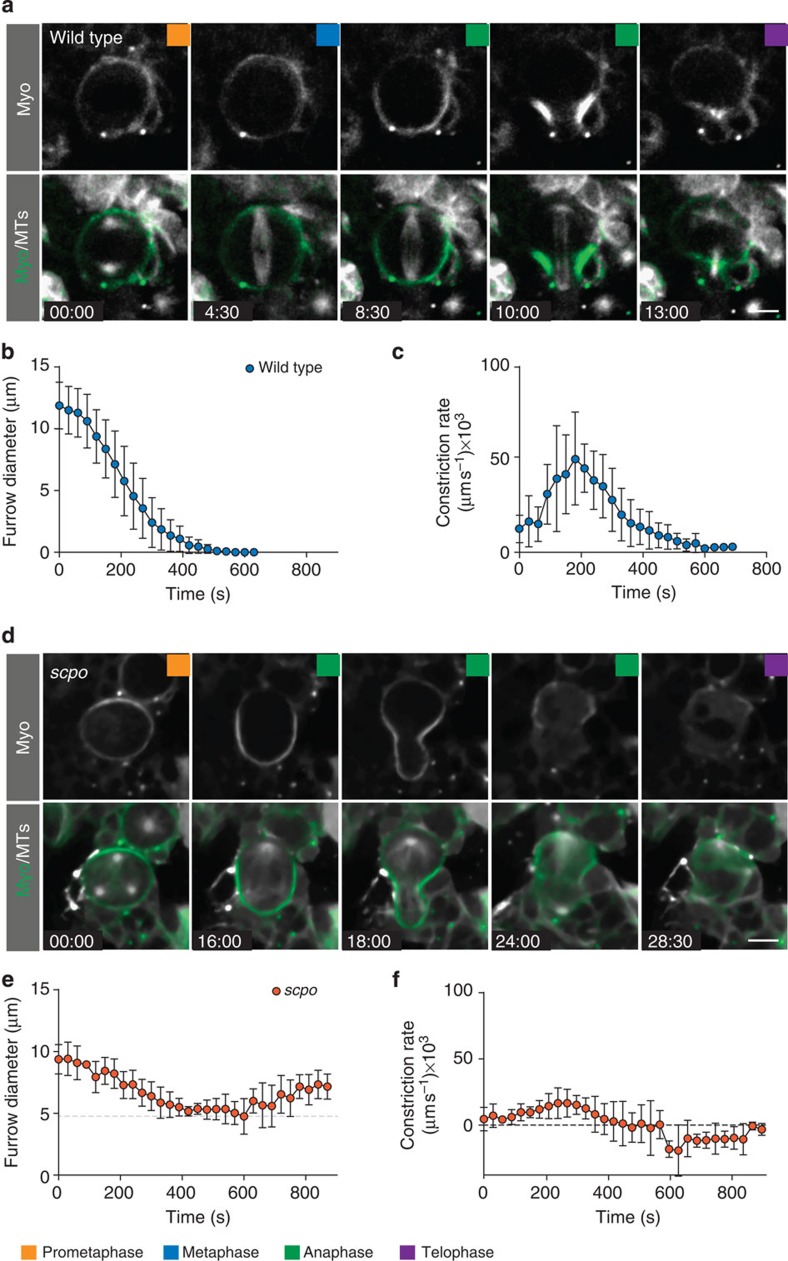
Survivin is required for cleavage furrow constriction. (**a**) Image sequence of a representative wild-type neuroblast expressing Sqh::GFP (green in overlay) and mCherry::Jupiter (white in overlay). (**b**) Quantification of wild-type neuroblast diameter measurements. (**c**) Constriction rates of wild-type neuroblasts. Averages and s.d. are shown (*n*=11). (**d**) Image sequence of a representative *scpo*^z2775^/*Df(3R)5780* mutant neuroblast expressing Sqh::GFP (green in overlay) and mCherry::Jupiter. (**e**) Diameter measurements. The grey dashed line indicates the minimal diameter. (**f**) Corresponding constriction rates of *scpo*^z2775^/*Df(3R)5780* mutant neuroblasts (*n*=8). Time in min:s; scale bar, 5 μm.

We performed live imaging on wild-type (Supplementary Movie 4) and *scpo*^*Z2775*^*/Df(3R)5780* (Supplementary Movie 5) mutant larval neuroblasts, expressing Sqh::GFP^27^ and mCherry::Jupiter,^11^ and first measured furrow constriction dynamics (see Methods). Using anaphase onset as a reference point, we found that wild-type neuroblasts completed constriction on average within 7.4 min (mean=464 s; s.d.=94 s; *n*=11; Fig. 5a,b; Table 1). We further calculated constriction rates and found that wild-type neuroblasts showed three distinct furrowing phases: constriction started slow, increased in speed and then slowed down again towards the end of cytokinesis (Fig. 5c). *scpo* mutant neuroblasts reached a minimal diameter of roughly 4.6 μm (s.d.=1.4 μm; *n*=8) on average in 8.4 min (mean=503 s; s.d.=118 s; *n*=8; Fig. 5d,e; Table 1) but then reopened the cleavage furrow. *scpo* mutant neuroblasts only contained a single slow constriction phase (Fig. 5f). We conclude that *scpo* is required for efficient cleavage furrow constriction and completion of cytokinesis. Furthermore, since *dlg*, *pins* single and *dlg;;pins* double mutants always complete cytokinesis (data not shown and ref. 11), Survivin’s role during cytokinesis seems distinct from the polarity-dependent cytokinesis pathway.

### The central spindle is required to complete furrowing

**Figure 6:**
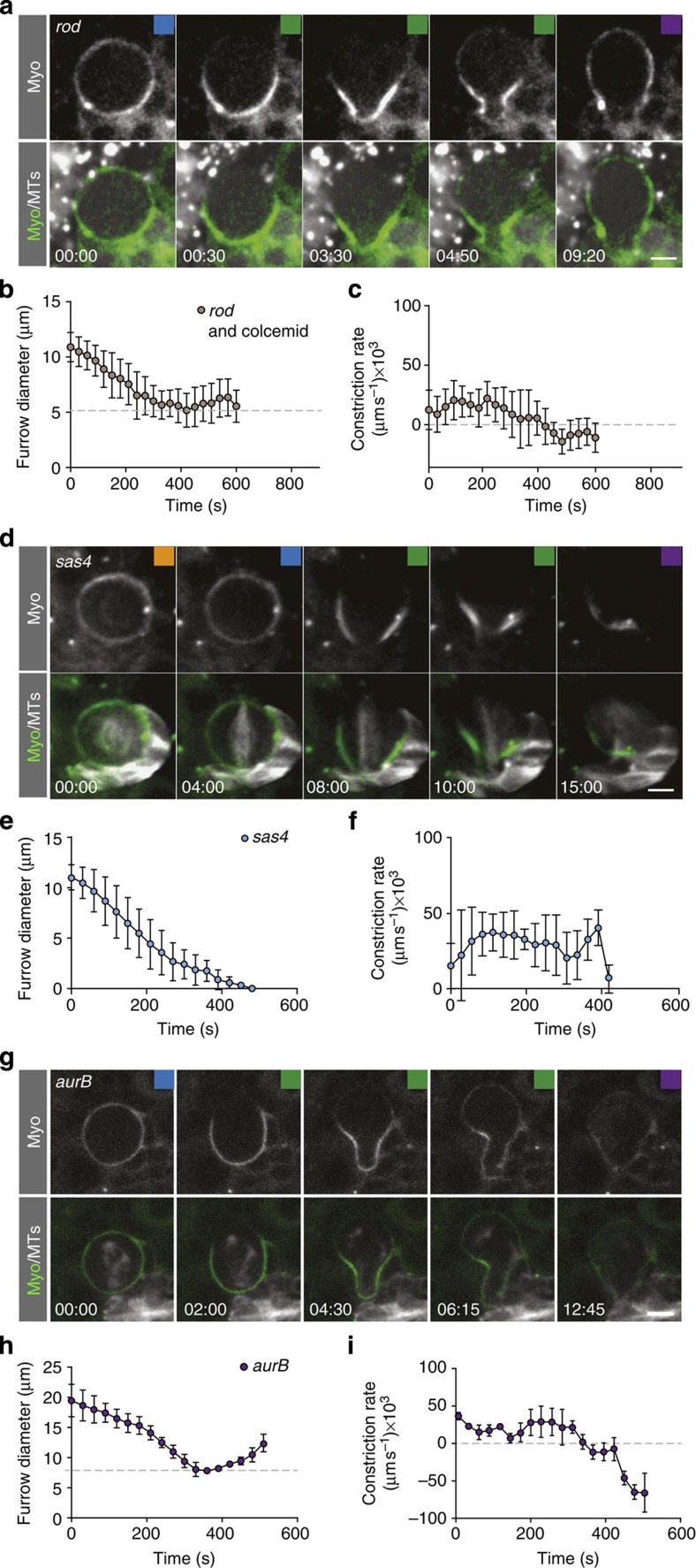
The mitotic spindle regulates furrow constriction through the CPC. (**a**) Image sequence of a representative *rod*^*H4.8*^ mutant neuroblast treated with colcemid, expressing Sqh::GFP (green in overlay) and mCherry::Jupiter (white in overlay). The mitotic spindle is completely depolymerized. (**b**) Diameter measurements of *rod*^*H4.8*^ and colcemid neuroblasts. The grey dashed line indicates the minimal diameter. (**c**) Corresponding constriction rates of *rod*^*H4.8*^ and colcemid neuroblasts. Averages and s.d. are shown (*n*=15). (**d**) Image sequence of a *sas4* mutant neuroblast, expressing Sqh::GFP (green in overlay) and mCherry::Jupiter (white in overlay). (**e**) Diameter measurements and (**f**) constriction rates of *sas4* mutant neuroblasts (*n*=10). (**g**) Image sequence of a representative neuroblasts, expressing RNAi against *aurB,* Sqh::GFP (green in overlay) and mCherry::Jupiter (white in overlay). (**h**) Diameter measurements and (**i**) constriction rates (*n*=4). Time in min:s; scale bar, 5 μm.

Failure to constrict could be due to central spindle defects since *scpo* mutants have been reported to perturb central spindle formation^18^. We analysed central spindle organization using kymographs, and found that wild-type neuroblast MTs bundled up during anaphase, but *scpo* mutant MTs appeared dispersed (data not shown). Thus, we asked whether the mitotic spindle is involved in cleavage furrow assembly, initiation, progression or completion of furrow constriction. To this end, we removed the mitotic spindle with colcemid in the spindle-assembly checkpoint mutant *rod* to allow entry into anaphase (see also above). We confirmed the previous result that *rod* mutant neuroblasts treated with colcemid and thus lacking the mitotic spindle entirely, show normal apical clearing of Myosin and initiate cleavage furrow constriction (Fig. 6a; refs 11, 15 and Supplementary Movie 6). Similar to *scpo* mutant neuroblasts, ingressing cleavage furrows failed to complete constricting and reopened during anaphase (Fig. 6a–c; Table 1).

We next asked whether astral MTs mediate constriction speed and completion by analysing *sas4* mutant neuroblasts, lacking functional centrosomes and astral MTs albeit central spindles are formed^28^. We found that in *sas4* zygotic mutant neuroblasts, the contractile ring formed normally, constricted and completed furrow ingression although constriction rate profiles are different compared with wild-type neuroblasts (Fig. 6d–f). For instance, we did not see the three distinct constriction phases but only observe a uniform constriction rate that slows down towards the end of cytokinesis (Figs 5b,c and 6e,f). Survivin is localized at the central spindle and the cleavage furrow in *sas4* mutants, albeit later than in wild-type neuroblasts (data not shown), but failed to reach the cortex in *rod* and colcemid-treated neuroblasts entirely (see above and Fig. 3b–d). In sum, we conclude that astral MTs are dispensable to complete cleavage furrow constriction, but the central spindle is absolutely essential to complete cytokinesis.

### AuroraB kinase is required for cleavage furrow constriction

Survivin is required for the correct localization of the CPC and *scpo* mutants fail to target AuroraB, the kinase of the CPC complex, to the spindle midzone^18^. Thus, we asked whether *survivin’s* cytokinesis phenotype is due to the diminished AuroraB (Ial/AurB) activity during anaphase. To this end, we knocked down AurB specifically in neuroblasts, expressing *aurB* RNAi with the neuroblast-specific worniuGal4 driver (worGal4; see also Methods)^29^. Under these conditions, we observed that most cells became highly polyploid after a few days of exposure to RNAi and failed to divide. We could thus only recover a few mitotic neuroblasts and found that these cells normally initiated cleavage furrow constriction but failed to complete contractile ring closure, similar to *scpo* and *rod* and colcemid conditions (Fig. 6g–i and Supplementary Movie 7). Similar to wild-type neuroblasts, we also found that Myosin was localized to the cortex and cleared on the apical side shortly after anaphase onset, indicating that initial Myosin relocalization is not perturbed. However, basal Myosin clearing seemed delayed. We conclude that AurB is required to complete contractile ring closure and that failure to complete constriction in *scpo* mutants is due to mislocalized AurB.

### The CPC mediates furrowing not just through centralspindlin

AurB has been shown to phosphorylate the centralspindlin complex component MKLP1 (refs 30, 31, 32) and Pav was reported to be abnormally localized in *scpo* mutant neuroblasts^18^. We analysed Pav::GFP localization with live cell imaging and measured Pav intensities at the constricting cleavage furrow and central spindle, respectively. In contrast to wild-type neuroblasts, we only detected weak Pav::GFP at the cleavage furrow and nothing at the central spindle in *scpo* mutant neuroblasts (Supplementary Fig. 5a–d and Supplementary Movies 8 and 9).

We next asked whether Pav is required for cleavage furrow ingression. We knocked down Pav using RNAi and found many large polyploid cells in third instar neuroblasts. Surprisingly, constriction defects were not the same as in *scpo*; *pav* RNAi-expressing neuroblasts were able to complete constriction, albeit with slower rates. Similarly, knocking-down *tumbleweed* (*tum/RacGAP50C*) showed the same phenotype; constriction was slow, but finally completed (Supplementary Fig. 5e,f). Nevertheless, many polyploid cells were found (data not shown). In contrast to wild-type neuroblasts, retaining Myosin in the midbody for several subsequent cell divisions, the midbody disappeared quickly in neuroblasts lacking Pav and Tum (data not shown). Thus, we speculate that polyploidy occurs because the fully constricted cleavage furrow fails to form a stable midbody, preventing subsequent abscission. Taken together, we conclude that the CPC is required for robust recruitment of the centralspindlin complex to the cleavage furrow, but that furrowing still occurs in the absence of the centralspindlin complex.

### Survivin is required to stabilize cleavage furrow positioning

**Figure 7:**
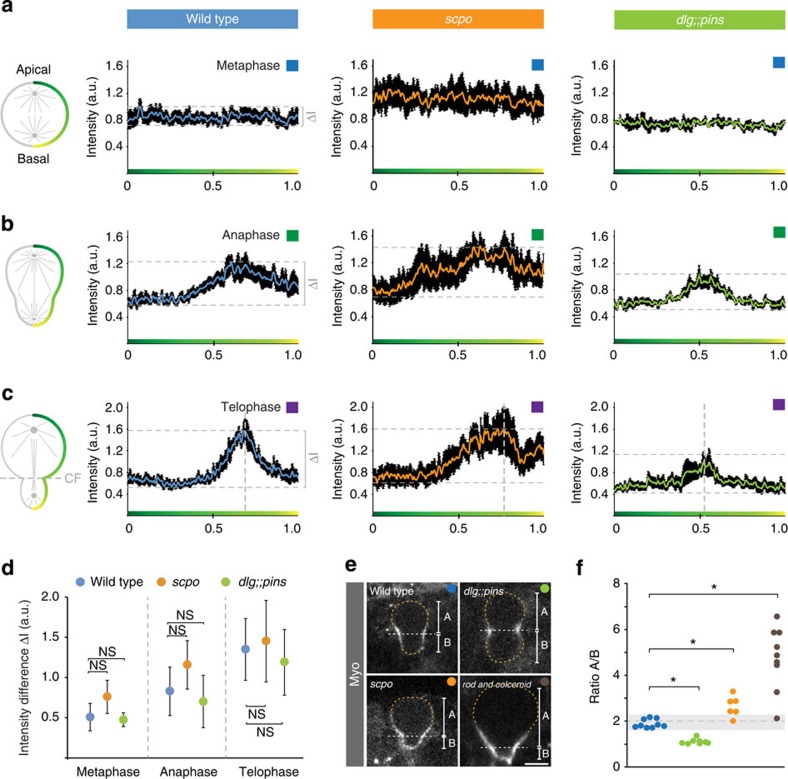
The spindle-dependent pathway stabilizes furrow positioning. Myosin intensity was measured along the neuroblast cortex (green gradient line) and averaged for wild-type, *scpo*^z2775^/*Df(3R)5780* and *dlg*^*m52*^*;;pins*^*P89*^ mutant neuroblasts at (**a**) metaphase, (**b**) anaphase and (**c**) telophase. Vertical dashed lines represent the forming cleavage furrow. Horizontal dashed lines indicate the difference between the lowest and the highest intensity values. These intensity differences are plotted in (**d**) for the indicated genotypes. Average values were derived from at least five neuroblasts. Error bars indicate s.d. (**e**) Cleavage furrow positioning was independently measured at the onset of furrowing for wild type (blue ball), *dlg;;pins* (green ball), *scpo* (orange ball) and *rod* and colcemid (brown ball). The A/B ratio (A, distance from the furrow to the apical cortex; B, distance from the furrow to the basal cortex) was plotted as a ratio in (**f**). Asterisk (*) denotes statistical significance. *P*=3.4 × 10^−9^ (two-sample equal variance *t*-test; wt vs *dlg;;pins*), *P*=0.00054 (two-sample equal variance *t*-test; wt vs *scpo*), *P*=0.00094 (two-sample unequal variance *t*-test; wt vs *rod* and colcemid). NS, not significant; *P*>0.01 (based on two-sample equal or unequal variance *t*-test). Dashed orange line outlines the cell boundaries. Dashed white line highlights the position of the cleavage furrow. Scale bar, 5 μm. wt, wild type.

Finally, we wanted to test whether Survivin affects Myosin dynamics and cleavage furrow positioning. To this end, we measured Myosin intensity along the neuroblast cortex (from apical to basal) and established intensity profiles (see Methods), allowing us to compare Myosin dynamics between wild-type and *scpo* mutant neuroblasts. Wild-type and *scpo* mutant neuroblasts showed comparable Myosin intensity and distribution during metaphase (Figs 5a,d and 7a). During anaphase, wild-type neuroblasts cleared Myosin from the apical cortex, enriching it in the furrow region (Figs 5a and 7b). *scpo* mutant neuroblasts also cleared Myosin apically but showed less precise Myosin accumulation in the furrow region by anaphase. Furthermore, basal Myosin clearing was delayed in *scpo* mutant neuroblasts (Figs 5d and 7b). Nevertheless, by telophase, *scpo* mutant neuroblasts also showed Myosin enrichment in the furrow region, comparable to wild type (Figs 5d and 7c). Similar Myosin dynamics were also observed for *aurB* RNAi-treated neuroblasts (Fig. 6g and data not shown). We quantified the intensity difference between the lowest and highest intensities at metaphase, anaphase and telophase, but failed to detect a significant difference between wild type and *scpo* mutants (Fig. 7d). Interestingly, *scpo* intensity profiles revealed that the furrow region is shifted towards the basal cortex (Fig. 7c). We performed the same analysis on *dlg;;pins* double mutants and found distinct intensity profiles: Myosin cleared at both poles at the same time, accumulating Myosin in the middle of the cell (Fig. 7b,c). However, intensity differences between the lowest and highest intensities at metaphase, anaphase and telophase were not significantly different in *dlg;;pins* double mutants compared with wild type (Fig. 7d).

We independently measured cleavage furrow positioning at the onset of furrowing in wild-type, *scpo* and *dlg;;pins* mutant neuroblasts and found that in both *scpo* and *dlg;;pins* mutants, the furrow position deviates from the wild-type scenario; *scpo* mutant neuroblasts shifted the furrow towards the basal cortex, whereas *dlg;;pins* mutant neuroblasts pushed it towards the apical cortex (Fig. 7e,f). This data suggests that the mitotic spindle is required to accurately position the cleavage furrow later in anaphase and telophase. Indeed, complete removal of the mitotic spindle using colcemid (in a *rod* mutant background) does not affect apical Myosin clearing^11^ but increased the basal furrow shift dramatically (Fig. 7e,f). Taken together, we conclude that Survivin and the mitotic spindle are dispensable for the initial establishment of asymmetric Myosin distribution but are required to stabilize the position of the polarity-induced basal cleavage furrow. Thus, the CPC and the mitotic spindle contribute towards accurate cleavage furrow positioning in late anaphase or early telophase.

## Discussion

With the identification of the polarity-dependent cleavage furrow-positioning pathway in *Drosophila* neuroblasts^11^,^15^ and spindle-independent cytokinesis mechanisms in other cells^12^,^13^,^14^, the function of the spindle-dependent pathway as well as the relative contribution of both pathways towards cytokinesis needs to be reassessed. To this end, a specific component of the spindle-dependent pathway has to be identified. The centralspindlin complex is not an ideal candidate since it is also partly controlled by the polarity-dependent pathway^11^ (and C. Cabernard, unpublished observations).

Here we report that the CPC component Survivin is a specific component of the spindle-dependent cytokinesis pathway in asymmetrically dividing *Drosophila* neuroblasts. Using *in vivo* live cell imaging of *Drosophila* neuroblasts in intact larval brains, we find that Survivin dynamically relocalizes from the kinetochores to the central spindle, the cleavage furrow and subsequently the midbody^10^,^17^. Photoconversion and FRAP experiments reveal that the Survivin pool at the central spindle and the cleavage furrow originates entirely from the chromosome-associated metaphase fraction. Thus, no new synthesis or recruitment from the cytoplasm contributes to the cleavage furrow population. Entry into the anaphase is necessary to release a significant fraction of Survivin from the centromere, and the mitotic spindle is required to transport this Survivin pool to the cleavage furrow, albeit astral microtubules are dispensable. Spindle rotation and manipulation experiments also show that Survivin’s cortical localization is independent of the polarity-dependent cytokinesis pathway. Thus, in contrast to the centralspindlin component Pavarotti, we find that Survivin’s localization entirely depends on the mitotic spindle but not the polarity-dependent pathway.

The identification of a specific component of the spindle-dependent cytokinesis pathway in neuroblasts allowed us to test the function of this pathway during asymmetric cell division. Using the separation-of-function allele *scpo*, only disrupting Survivin’s function and CPC localization from anaphase onwards^18^, we show that Survivin plays an important role in MT bundling and cleavage furrow constriction. This phenotype is entirely mimicked by neuroblasts devoid of the mitotic spindle; furrow ingression is slowed down and constriction fails to complete. Since under these circumstances the CPC fails to relocalize to the central spindle and the cleavage furrow, we can conclude that in asymmetrically dividing neuroblasts, the mitotic spindle controls completion of contractile ring closure through the CPC. In agreement with these conclusions is the finding that knockdown of *aurB*, the kinase entity in the CPC complex, results in a very similar phenotype.

What are the CPC targets involved in the completion of contractile ring closure? A prime candidate is the centralspindlin complex, since its localization depends on the CPC^30^,^31^,^32^. In agreement with a previous report, we find that Pav is mostly delocalized in *scpo* mutants. Furthermore, knockdown of *pav* still allows cleavage furrow constriction. Similar results were also obtained for the second centralspindlin component, *tumbleweed*. Since we see very strong spindle organization defects and polyploidy, we do not think that the lack of a strong furrowing phenotype is owing to partial inactivation of the respective gene products, but favour the hypothesis that these proteins are acting redundantly with other CPC targets. For instance, Septins, membrane-associated filamentous GTP-binding proteins implicated in cytokinesis^33^, have recently been shown to genetically interact with *aurB*^34^. Furthermore, the lack of Tum and Pav could also compromise the stability of the midbody. Indeed, whereas Myosin stably labels the midbody for many cell divisions in wild-type neuroblasts, Myosin quickly disappears from the newly formed midbody in *tum*-or *pav*-deficient neuroblasts (data not shown).

We also found that Survivin and the mitotic spindle contribute towards accurate cleavage furrow positioning; in *scpo* mutants—and also neuroblasts lacking the mitotic spindle completely—the cleavage furrow is shifted towards the basal cortex. It is important to note that this phenotype is different from mutants compromising the polarity-dependent pathway, in which the cleavage furrow is shifted towards the apical neuroblast cortex, mispositioning it in the middle of the cell^11^. Thus, we conclude that accurate basal furrow positioning also requires a spindle-dependent cue in late anaphase/early telophase. This is not entirely surprising since spindle rotation experiments, uncoupling the two pathways, resulted in the formation of two cleavage furrows^11^.

The nature of the spindle-dependent cue and the mechanism for accurate furrow positioning are currently unclear, but several hypotheses can be formulated: similar to *Drosophila* Schneider cells^35^, Myosin filaments could be disassembled at the apical and then the basal pole and *de novo* reassembled from soluble dimers through Rho1 induction at the neuroblast furrow region. Alternatively, the polarity-dependent pathway could induce a basal directed Myosin flow, and stabilized through the CPC and downstream effectors at the forming cleavage furrow. Since Survivin reaches the furrow region at the onset of furrowing, this scenario is plausible. Last but not least, in addition to the polarity-dependent Myosin flow, an apical-directed Myosin flow, originating on the basal cortex, could be induced through the CPC and the mitotic spindle. Indeed, in *scpo* and colcemid-treated neuroblasts, we see a delay in basal Myosin clearing (see also ref. 11). These hypotheses are currently being tested.

**Figure 8:**
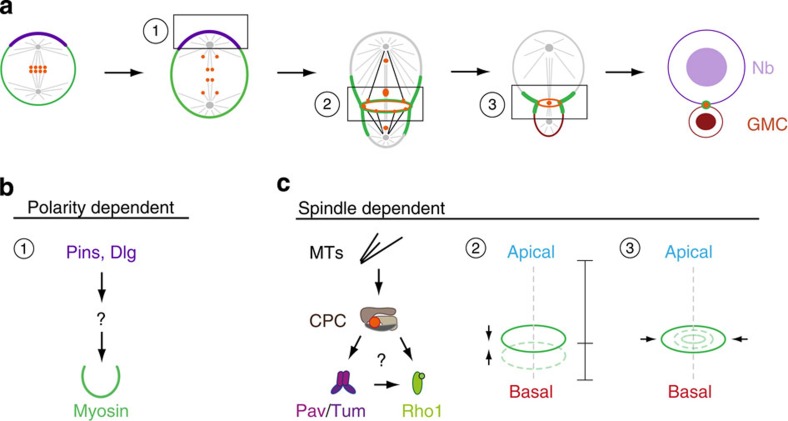
Model. (**a**) Schematic representation of Survivin’s (orange) dynamic redistribution during asymmetric cell division. By metaphase, the entire Survivin pool is associated with chromosomes and lines up at the metaphase plate. After anaphase onset, a fraction of this Survivin pool is loaded onto microtubules and redistributed to the central spindle as well as the forming furrow region, which is shifted towards the basal cortex. Microtubules decorated with Survivin (black lines), contact the cortex at the cleavage furrow, forming a Survivin ring (orange ring) just inside the Myosin-containing contractile ring (green ring). This Survivin ring lines up with the Survivin pool at the central spindle during late anaphase/telophase, and both pools contribute to the forming midbody. (**b**) Two pathways with distinct functions control the generation of physical and molecular asymmetry in *Drosophila* neuroblasts. (**b**) (1) The polarity-dependent pathway induces asymmetric Myosin localization via Pins and Dlg shortly after anaphase onset. The molecular composition of this pathway is not known. Asymmetric Myosin localization is required for basal cleavage furrow positioning. (**c**) The spindle-dependent pathway not only stabilizes the basal furrow position (2) but also controls cleavage furrow ingression and completion (3). The vertical dashed line indicates the polarity/division axis. Both pathways act spatially and temporally separate to generate a large, self-renewed neuroblast and a differentiating small GMC. The mitotic spindle is responsible to relocalize the CPC, which activates the centralspindlin complex (Pav/Tum). Whether the centralspindlin complex maintains Myosin constriction through Rho1 is unclear in this system. Other CPC targets could act redundantly to complete cytokinesis. Nb, neuroblast; GMC, ganglion mother cell.

Taken together, the results reported here not only corroborate the recent finding that asymmetrically dividing neuroblasts contain two cytokinesis pathways^11^ but also that they are spatially and temporally separated. It is thus the timely interplay between the two pathways that ultimately ensures that the cleavage furrow is formed at the correct position. Our phenotypic characterization is inconsistent with the idea that the two pathways are simply acting redundantly and that the polarity-dependent pathway serves as a backup cytokinesis pathway. On the basis of earlier results and our findings here, we thus propose that *Drosophila* neuroblasts utilize the polarity-dependent pathway, composed of the polarity proteins Pins and Dlg, to induce asymmetric Myosin localization during early anaphase^11^,^15^. The spindle-dependent pathway subsequently engages the CPC, but possibly also other molecules, to stabilize the position of the furrow in late anaphase/early telophase and to complete cytokinesis^11^,^15^ (Fig. 8). In the future, it will be important to elucidate the mechanism by which the CPC and the mitotic spindle regulate accurate furrow positioning. Furthermore, the regulatory mechanisms ensuring the timely interplay between the polarity and spindle pathway remain to be investigated.

## Methods

### Fly strains and genetics

All mutant chromosomes were balanced over FM7 actin::GFP or TM6B, Tb. The following alleles and deficiency stocks were used: *scpo*^*z2775*^ (ref. 18), *Df(3R)5780* (ref. 18) (a deficiency removing the entire *survivin* gene), *dlg*^*m52*^ (ref. 36), *pins*^*P89*^ (ref. 37), *mud*^*4*^ (ref. 38) and *DSas4*^*M*^, *DSas4*^*l(3)S2214*^ (ref. 28), *rod*^*H4.8*^ (ref. 20). Mutants were either analysed in a homoallelic combination or over the corresponding deficiency. Transgenes and fluorescent markers: *worGal4, pUAST-cherry::Jupiter*^2^, *sqh::GFP*^27^, *UAS-GFP:PavNLS5* (ref. 39), *survivin::GFP*^18^, *survivin::mDendra2* (this work), *His2A::mRFP1*^40^, *UAS-ALD-Survivin::EGFP* (this work). UAS transgenes were expressed using *worGal4* (ref. 29). RNAi lines to knockdown *pav*, *aurB* and *tum* were obtained from the Vienna Drosophila RNAi Center or the Bloomington Stock Center. To minimize unspecific phenotypes upon *aurB* knockdown, we kept *aurB* RNAi-expressing larvae at 18 °C to minimize expression of *aurB* antisense RNA. Subsequent dissections and imaging was performed at RT.

*scpo* mutants were incubated at 29 °C for up to 20 h prior to imaging. Imaging was also performed at 29 °C for most experiments.

### Generation of *survivin::mDendra2* construct

*survivin’s* coding region, its 5′ and 3′untranslated regions and *mDendra2* (ref. 41) were PCR amplified and cloned into the BamHI and KpnI sites of the pattB transformation vector using In-Fusion technology (Takara, Clontech). The resulting construct was injected into attP VK00033 and VK00022, respectively.

### Generation of *ALD::EGFP::Survivin* construct

*EGFP::Survivin* was PCR amplified and subcloned into MIuI and KpnI sites of pUAST-attB-ALD (A. Tsankova, unpublished) using In-Fusion technology (Takara, Clontech). The resulting construct was injected into attP40 and VK0005 (Genetic Services).

### Live imaging sample preparation

Live imaging samples were prepared according to a recently published protocol^42^. In brief: S2-media (Invitrogen) was supplemented with 10% PBS, 10%, PenStrepNeo 2%, Insulin 0.02 mg ml^−1^, Glutamine 20 mM, Glutathione 0.04 mg ml^−1^, and 20-Hydroxyecdysone 5 μg ml^−1^ according to a recently published report^43^. The 96-h after egg-laying (AEL)-old mutant or wild-type larval brains were dissected in imaging medium. The dissected brains were transferred onto a gas-permeable membrane (YSI Life Sciences 5793), fitted on a metallic slide. Brains were oriented with the brain lobes facing the coverslip. Excess media was removed until the brain lobes were in contact with the coverslip. The sample was sealed with Vaseline.

Colcemid experiment: shortly before mounting the brains, the drug was added to supplemented S2-media at a final concentration of 5 μg ml^−1^.

Live samples were imaged with an Andor revolution spinning disc confocal system, consisting of a Yokogawa CSU-X1 spinning disk unit and two Andor iXon3 DU-897-BV EMCCD cameras. A 60X/1.4NA oil immersion objective mounted on a Nikon Eclipse Ti microscope was used for most images. Images contain voxel sizes of 0.22 × 0.22 × 0.5 μm ( × 60, 1.4 NA spinning disc).

Temperature shift experiments for *scpo* mutants were performed on a Perkin Elmer spinning disc microscope, equipped with two Hamamatsu C9100-50 frame transfer EMCCD cameras and a sealed chamber allowing to image at 29 °C. A × 63, 1.4NA objective, mounted on a Leica DM6000 was used.

### Photoactivation

The 96-h AEL larval brains expressing one to three copies of *survivin::mDendra2* were used. Photoconversion experiments were performed on an Andor Revolution spinning disc system containing the FRAPPA unit (Andor). Several regions of interests (ROIs) were manually chosen in the GFP channel. Survivin at the metaphase plate was irradiated before anaphase onset. Before photoconversion, single Z planes containing ROIs were scanned for ten time points with maximum speed. Subsequently, ROIs were irradiated with the 405 nm laser line (9.7%; 20 repeats; 50 μs dwell time). After photoconversion, the entire neuroblast was scanned with a z-step size of 0.65 μm. Photoconverted Survivin-mDendra2 emits red fluorescence. Converted and unconverted mDendra2 emission were merged in Andor IQ2 and converted into Imaris.

### FRAP

The 96-h AEL larval brains expressing *survivin::GFP* and *mCherry::Jupiter* were used. Several ROIs were manually chosen in the GFP channel. Before FRAP, single Z planes containing ROIs were scanned for ten time points with maximum speed using 300-ms exposure time. Subsequently, the 488-nm laser line was used to irradiate ROIs (100%; 25 repeats; 50 μs dwell time) two times in short succession (~3 s pause between irradiations). After FRAP, the entire neuroblast was scanned with a z-step size of 0.65 μm.

### Antibodies

The following primary antibodies were used: rabbit anti-Aurora B (1:500; gift from Régis Giet), rabbit anti-Survivin (1:250; gift from Maria Grazia Giansanti), rat anti-αTubulin (1:500; Serotec). Secondary antibodies were from Molecular Probes and the Jackson Immuno Laboratory.

For three-dimensional structured illumination microscopy (3D-SIM), the following primary antibodies were used: mouse anti-αTubulin (1:2500; Sigma, DM1A) and rat anti-Miranda (1:1000; gift from Chris Doe). The following secondary antibodies were used: goat anti-mouse-568 (1:500; Molecular Probes) and donkey anti-rat Alexa Fluor 647 (1:300; Jackson Immuno).

### Immunohistochemistry

Larval brains were dissected in Schneider’s insect medium and fixed for 20 min in 4% paraformaldehyde in PEM (100 mM PIPES pH 6.9, 1 mM EGTA and 1 mM MgSO_4_). After fixing, the brains were washed with PBSBT (1 × PBS, 0.1% Triton-X-100 and 1% BSA) and then blocked with 1 × PBSBT for 1 h. Primary antibody dilution was prepared in 1 × PBSBT, and brains were incubated overnight or during 2 days at 4 °C. Brains were washed with 1 × PBSBT three times for 30 min each and then incubated with secondary antibodies diluted in 1 × PBSBT at 4 °C, overnight. The next day, brains were washed with 1 × PBST (1 × PBS, 0.1% Triton-X-100) three times for 30 min each and kept in Vectashield (Vector Laboratories) mounting media at 4 °C. For super resolution, brains were kept in PBS, then mounted in Vectashield shortly before acquisitions.

### Super-resolution 3D-SIM

3D-SIM was performed on a microscope system (DeltaVision OMX-Blaze version 4; Applied Precision, Issaquah, WA) equipped with 405, 445, 488, 514, 568 and 642 nm solid-state lasers. Images were acquired using a Plan Apo N × 60, 1.42 NA oil immersion objective lens (Olympus) and four liquid-cooled sCMOs cameras (pco Edge, full frame 2560 × 2160; Photometrics). Exciting light was directed through a movable optical grating to generate a fine-striped interference pattern on the sample plane. The pattern was shifted laterally through five phases and three angular rotations of 60° for each z section. Optical z-sections were separated by 0.125 μm. The laser lines 488, 568 and 642 nm were used for 3D-SIM acquisition. Exposure times were typically between 3 and 100 ms, and the power of each laser was adjusted to achieve optimal intensities of between 5,000 and 8,000 counts in a raw image of 15-bit dynamic range at the lowest laser power possible to minimize photobleaching. Multichannel imaging was achieved through sequential acquisition of wavelengths by separate cameras.

Raw 3D-SIM images were processed and reconstructed using the DeltaVision OMX SoftWoRx software package (Applied Precision^44^). The resulting size of the reconstructed images was of 512 × 512 pixel from an initial set of 256 × 256 raw images. The channels were aligned in the image plane and around the optical axis using predetermined shifts as measured using a target lens and the SoftWoRx alignment tool. The channels were then carefully aligned using alignment parameter from control measurements with 0.5 μm diameter multispectral fluorescent beads (Invitrogen, Molecular Probes).

### Image processing and calculations

Images were processed using Imaris × 64 7.5.2 and FiJi. Andor IQ2 files were converted into Imaris using custom-made Matlab codes. Kymographs, made in ImageJ, were used to analyse and compare Survivin behaviour in wild type and mutants. For the *scpo*^*z2775*^ mutant, custom-made Matlab codes were used to calculate the rate of ingression at the cleavage furrow. We used ImageJ/Fiji to establish intensity profiles, measuring Sqh::GFP intensities along a line from the apical to the basal cortex. Starting at metaphase, each time point was measured for at least six cells and then averaged.

Pictures were cropped in Corel Draw Photo or Photoshop and assembled in Corel Draw or Illustrator. Quantifications and graphical representations were generated in Microsoft Excel, Numbers and Graphpad Prism.

### Statistics and sample number

Whenever necessary, F-tests were performed to determine the variances of groups to be compared. If *P*<0.05 in the F-test, we performed the two-sample unequal variance *t*-test. For *P*>0.05, we applied the two-sample equal variance *t*-test. For each experiment, the data was collected from at least three independent brain lobes.

## Additional information

**How to cite this article:** Roth, M. *et al.* Asymmetrically dividing *Drosophila* neuroblasts utilize two spatially and temporally independent cytokinesis pathways. *Nat. Commun.* 6:6551 doi: 10.1038/ncomms7551 (2015).

## Supplementary Material

Supplementary Figures Supplementary Figures 1-5

Supplementary Movie 1 Survivin dynamically relocalizes during mitosis. Wild type larval neuroblast expressing Survivin::GFP (green in merged movie on the left; white, single channel movie on the right) and the cortex marker Sqh::mCherry (white in merged movie on the left). The neuroblast was imaged every 30 s. Time scale is h:mm:ss. The scale bar is 5 μm.

Supplementary Movie 2 Kinetochore associated Survivin splits up into three Survivin pools. Wild type larval neuroblast expressing Survivin::GFP (green in merged movie on the left; white, single channel movie on the right) and the DNA marker His2A::mRFP1 (white in merged movie on the left). The neuroblast was imaged every 20 s. Time scale is h:mm:ss. The scale bar is 5 μm.

Supplementary Movie 3 Survivin is not sufficient to recruit Myosin and to induce ectopic furrowing. Wild type larval neuroblast expressing ALD-Survivin::EGFP (green in merged movie on the left and the single channel movie in the middle) and Sqh::mCherry (white in merged movie on the left and the single channel movie on the right). The neuroblast was imaged every 30 s. Time scale is h:mm:ss. The scale bar is 5 μm.

Supplementary Movie 4 Cleavage furrow formation and ingression in wild type neuroblasts. Wild type larval neuroblast expressing Sqh::GFP (green in merged movie on the left; white, single channel movie on the right) and the spindle marker mCherry::Jupiter (white in merged movie on the left). The neuroblast was imaged every 30 s. Time scale is h:mm:ss. The scale bar is 5 μm.

Supplementary Movie 5 Cleavage furrow ingression is compromized in *scpo* mutant neuroblasts. Representative scpo mutant neuroblast expressing Sqh::GFP (green in merged movie on the left; white, single channel movie on the right) and the spindle marker mCherry::Jupiter (white in merged movie on the left). The neuroblast contains multiple centrosomes. The neuroblast was imaged every 45 s. Time scale is h:mm:ss. The scale bar is 5 μm.

Supplementary Movie 6 MTs are required to complete cytokinesis. *rod^H4.8^* mutant neuroblast treated with colcemid, expressing the cortex marker Sqh::GFP (green in merged movie on the left; white, single channel movie on the right) and mCherry::Jupiter (white in merged movie on the left). Note that the mitotic spindle is completely depolymerized. The neuroblast was imaged every 10 s. Time scale is h:mm:ss. The scale bar is 5 μm.

Supplementary Movie 7 AurB is required to complete cytokinesis. *aurB RNAi* treated neuroblast, expressing the cortex marker Sqh::GFP (green in merged movie on the left; white, single channel movie on the right) and mCherry::Jupiter (white; left movie). Note that this neuroblasts contains multiple centrosomes. The neuroblast was imaged every 15 s. Time scale is h:mm:ss. The scale bar is 5 μm.

Supplementary Movie 8 Pav dynamically relocalizes to the cleavage furrow. Wild type neuroblast, expressing the centralspindlin marker Pav::GFP (green in merged movie on the left; white, single channel movie on the right) and mCherry::Jupiter (white; left movie). The neuroblast was imaged every 30 s. Time scale is h:mm:ss. The scale bar is 5 μm.

Supplementary Movie 9 Pav localization is compromized in scpo mutant neuroblasts. Representative *scpo* mutant neuroblast expressing Pav::GFP (green in merged movie on the left; white, single channel movie on the right) and the spindle marker mCherry::Jupiter (white in merged movie on the left). The neuroblast is already binucleate due to failed cytokinesis in a previous cell cycle. The neuroblast was imaged every 28.3 s. Time scale is h:mm:ss. The scale bar is 5 μm.

## Figures and Tables

**Table 1:** Constriction parameters of wild-type and mutant conditions.

	**Minimal diameter (μm)**		**Time until minimal diameter (s)**		**Average constriction rates (nm** **s**^−1^**)**		***n***
	**Mean**	**s.d.**	**Mean**	**s.d.**	**Mean**	**s.d.**	
Wild type	0	0	464	93.5	27.01	7.16	11
*scpo*	4.59	1.41	502.5	117.9	14.95	7.49	8
*rod* and *colcemid*	4.51	1.53	362	88.3	17.77	7.84	15
*sas4*	0	0	372	67.35	30.47	5.55	10
*aurB* RNAi	8.99	4.39	345	96.05	25.72	10.18	4
