# Supplementary material for: Asymmetrically dividing Drosophila neuroblasts utilize two spatially and temporally independent cytokinesis pathways
Source: Nat Commun. 2015 Mar 20;6:6551. doi: 10.1038/ncomms7551 (PMC4544045; doi:10.1038/ncomms7551)
Supplement: Supplementary Figures — 1-5 [file ncomms7551-s1.pdf]

SUPPLEMENTARY INFORMATION

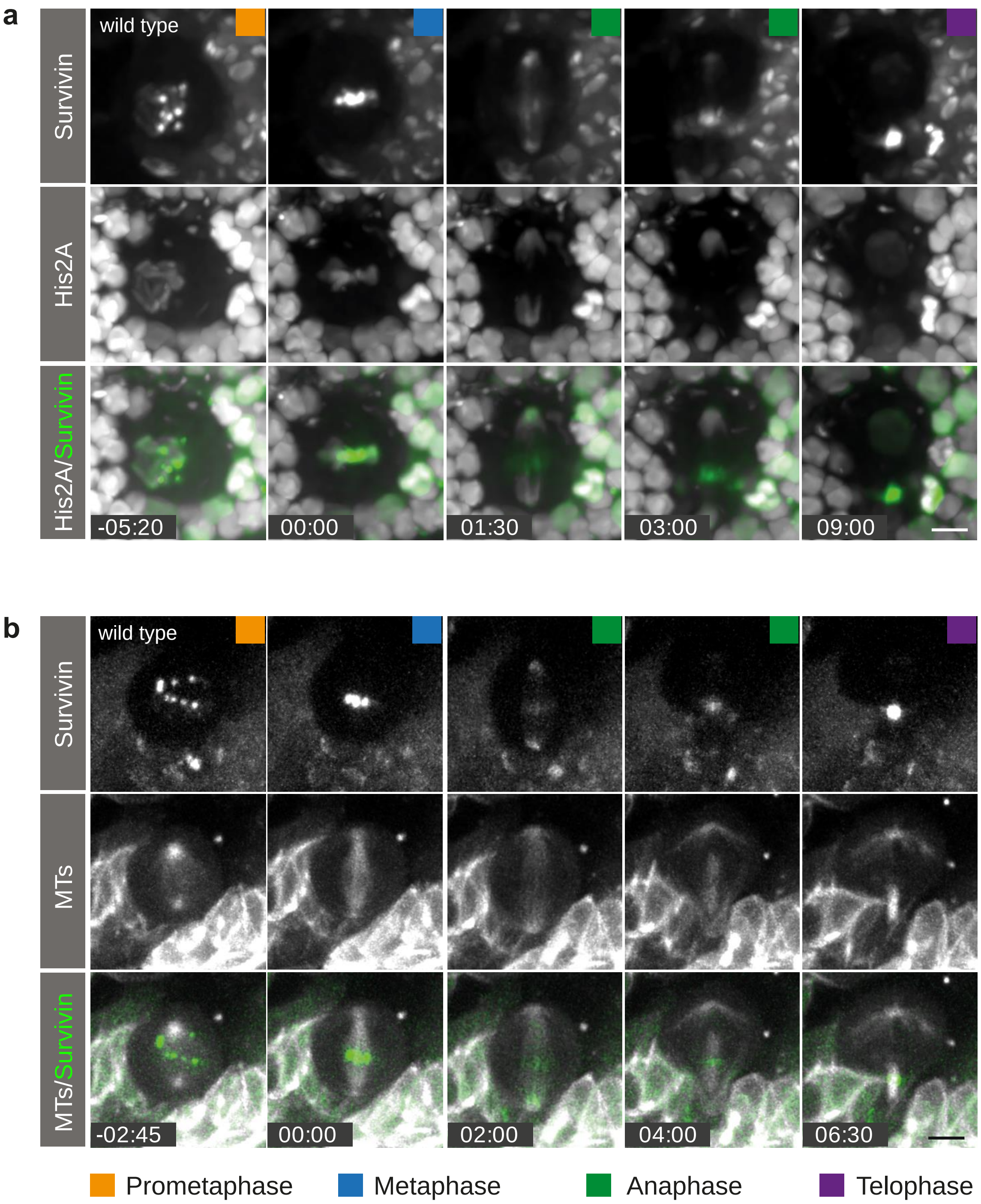

Supplemental Figure 1: Dynamics of Survivin relocalization in *Drosophila* neuroblasts.

(a) Image sequence of a representative wild type neuroblast expressing Survivin::GFP (green in overlay; white in single channel) and His2A::mCherry (white in overlay and single channel). **(b)** Survivin::GFP (green in overlay; white in single channel) and mCherry::Jupiter (white in overlay and single channel). MTs = Microtubules. Time in min:sec; scale bar is 5µm.

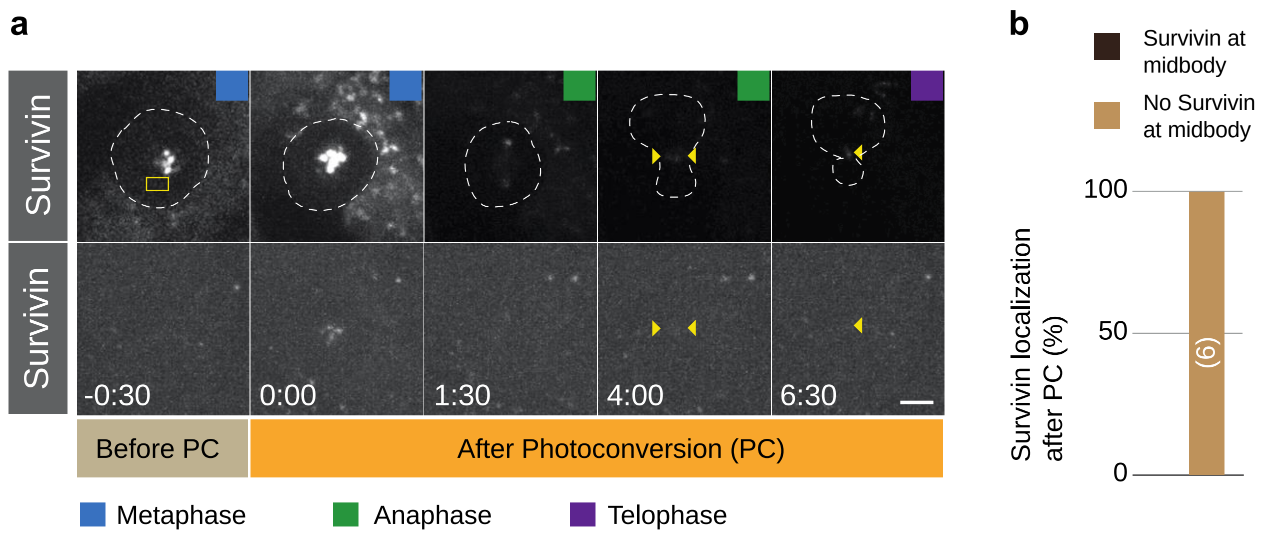

Supplemental Figure 2: Cytoplasmic Survivin at metaphase does not contribute to midbody-associated Survivin at telophase.

(a) Photoconversion control experiment. The image sequence shows a representative wild type neuroblasts expressing Survivin::mDendra2. Photoconversion was performed in the cytoplasm (yellow box) of metaphase neuroblasts. No photoconverted Survivin can be detected at the midbody in telophase. **(b)** Quantification of control experiments. Time in min:sec; scale bar is 5μm.

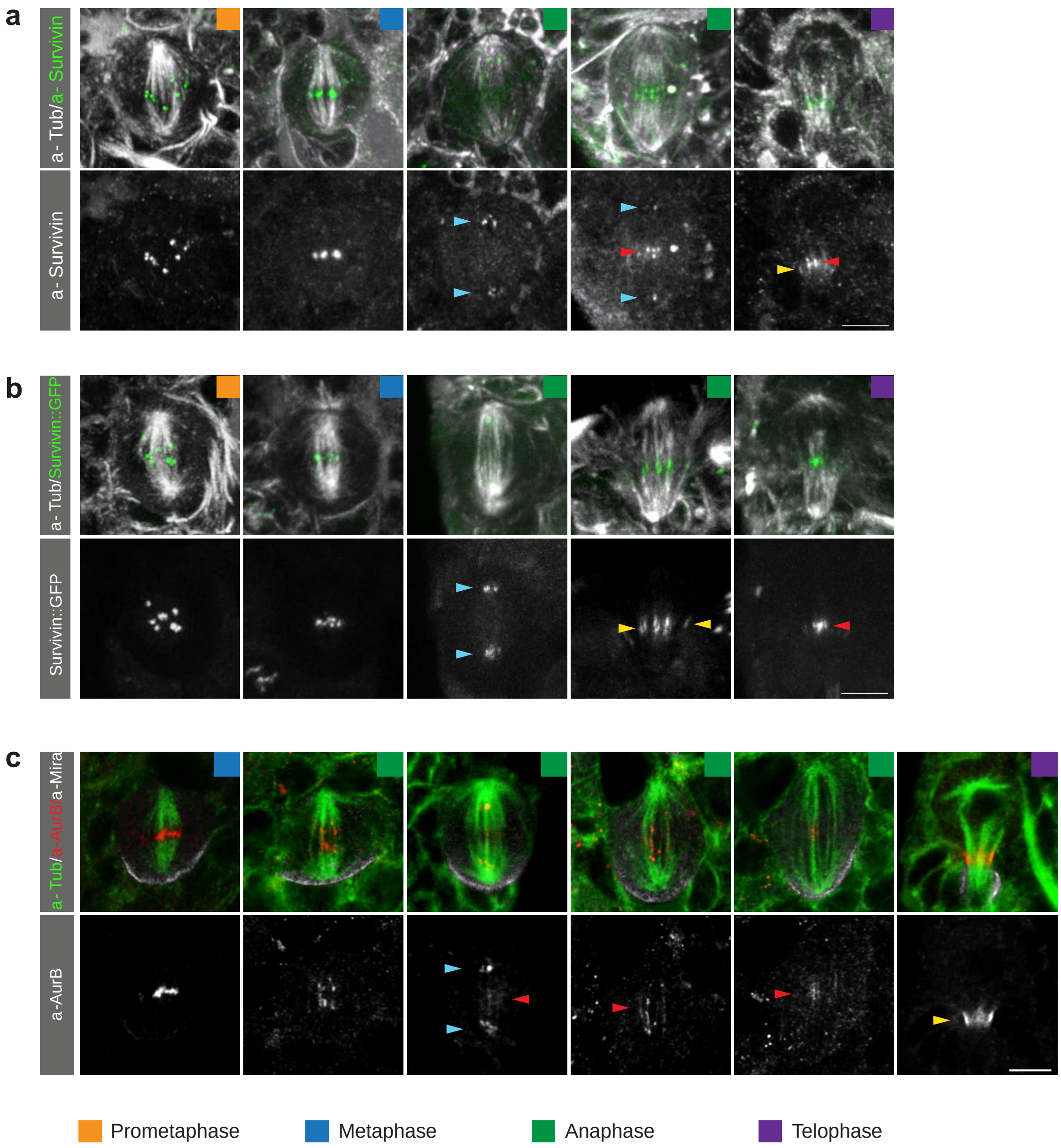

Supplemental Figure 3: Survivin::EGFP accurately reflects endogenous Survivin and AurB localization.

(a) Wild type third instar larval brain neuroblasts stained with α-tubulin (white) and α-Survivin (green in overlay, white in single channel below). **(b)** Wild type larval brain neuroblast expressing Survivin::EGFP and stained with α-tubulin (white) and α-GFP (green in overlay, white in single channel below). **(c)** Wild type neuroblasts stained with α-tubulin (green), α-Miranda (Mira; white in overlay) and α-AuroraB (red in overlay, white in single channel below). In all panels, colored arrowheads highlight Survivin and AuroraB localized at separating chromosomes (blue arrowheads), the central spindle (red arrowheads) and the cleavage furrow (yellow arrowheads). Mitotic stages are indicated with colored boxes. Scale bar is 5μm.

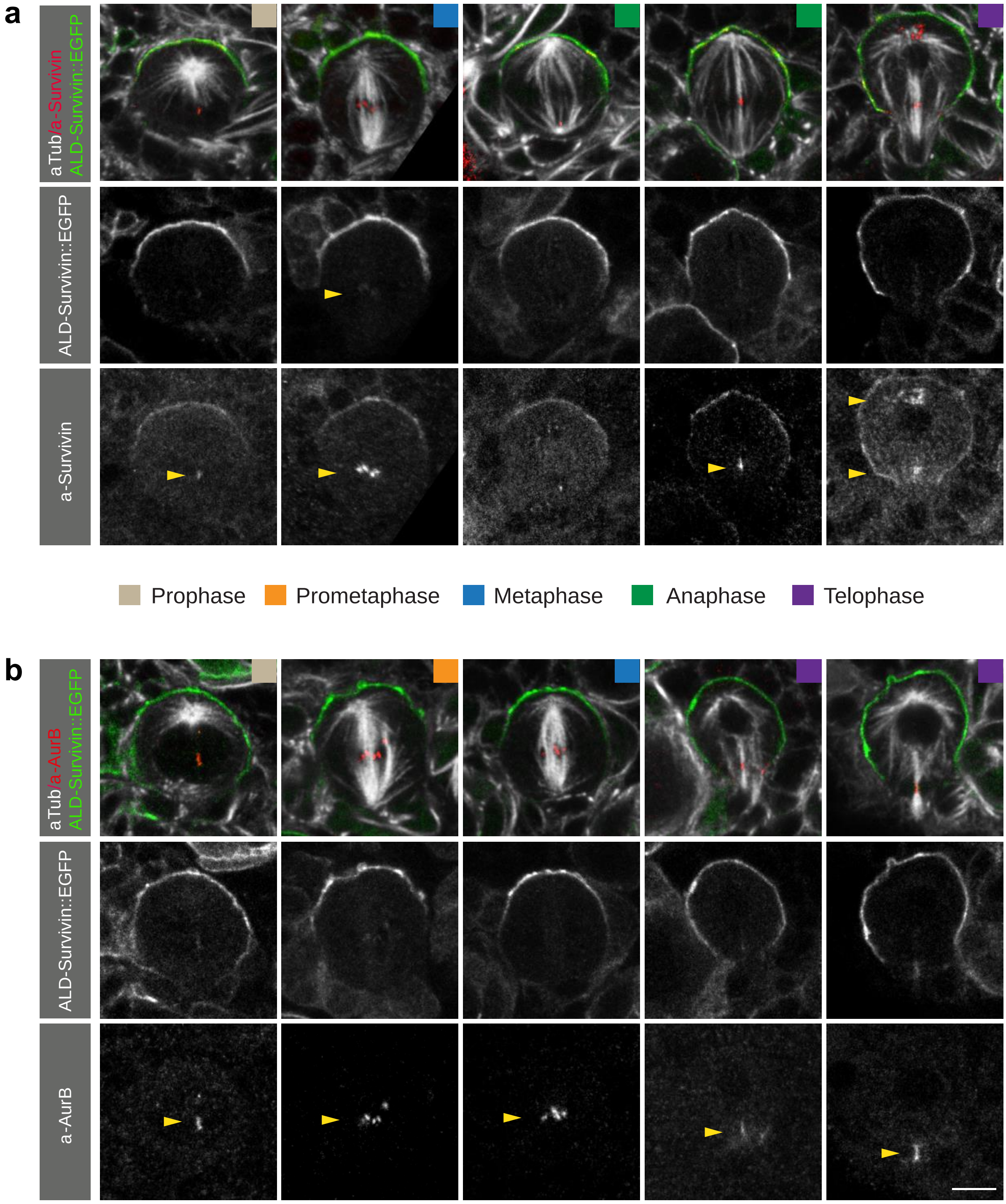

Supplemental Figure 4: ALD-Survivin::EGFP ectopically localizes Survivin to the apical neuroblast cortex but is not sufficient to recruit AuroraB.

(a) Wild type neuroblasts expressing ALD-Survivin::EGFP and stained with α-tubulin (white in overlay) and α-Survivin (red in overlay, white in single channel below) or **(b)** α-AuroraB (red in overlay, white in single channel below). A small portion of ALD-Survivin::EGFP also colocalizes with untagged endogenous Survivin (yellow arrowheads). AuroraB is not recruited to the apical domain. Scale bar is 5μm.

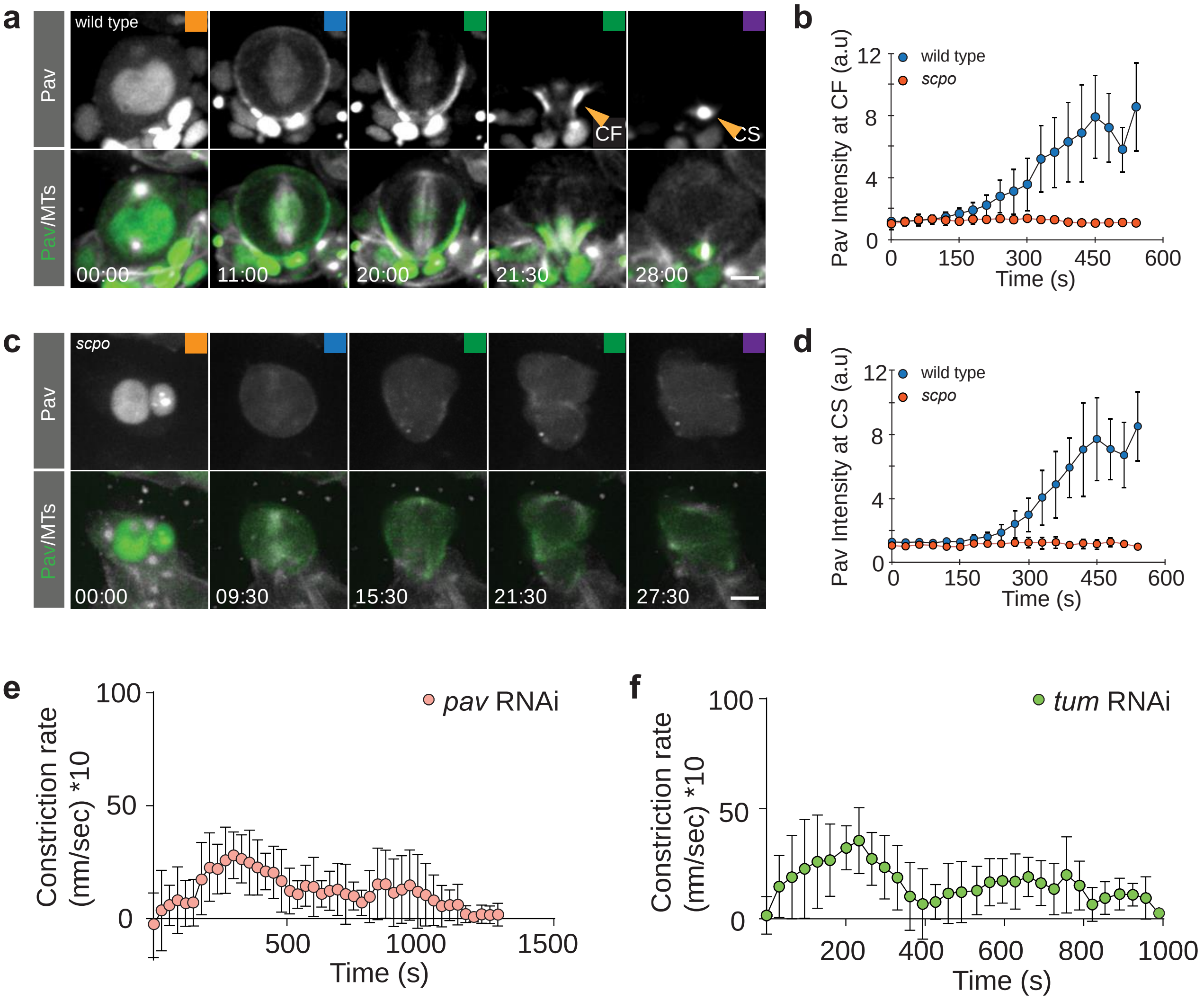

Supplemental Figure 5: CPC's cleavage furrow constriction is not just mediated through the centralspindlin complex.

(a) Image sequence showing a representative wild type neuroblasts expressing Pav::GFP (green in overlay) and mCherry::Jupiter (white in overlay). Pav's localization to the cleavage furrow (CF) and central spindle (CS) is highlighted with a yellow arrowhead. **(b)** Pav::GFP intensity measurements at the CF in wild type (blue) and *scpo* (red) (n = 7). **(c)** Pav::GFP localization in *scpo* mutant neuroblasts (n = 4). The neuroblast is binucleate due to failed cytokinesis in the previous cell cycle. **(d)** Pav::GFP intensity measurements at the CS in wild type (blue) and *scpo* (red). The plot shows the average intensity ratios between Pav::GFP at the CF or CS and cytoplasmic Pav::GFP. Error bars correspond to standard deviation (SD). Cleavage furrow constriction rates of neuroblast expressing RNAi against *pav* (n = 8) **(e)** and *tum* (n = 10) **(f)**. Averages and standard deviation are shown.
